# Phonetic and phonological imitation of intonation in two varieties of Italian

**DOI:** 10.3389/fpsyg.2014.01226

**Published:** 2014-11-04

**Authors:** Mariapaola D’Imperio, Rossana Cavone, Caterina Petrone

**Affiliations:** ^1^CNRS, LPL, UMR 7309, Aix-Marseille UniversitéAix-en-Provence, France; ^2^Institut Universitaire de FranceParis, France

**Keywords:** imitation, phonetic convergence, Neapolitan Italian, Bari Italian, intonation, tonal alignment, tonal scaling

## Abstract

The aim of this study was to test whether both phonetic and phonological representations of intonation can be rapidly modified when imitating utterances belonging to a different regional variety of the same language. Our main hypothesis was that tonal alignment, just as other phonetic features of speech, would be rapidly modified by Italian speakers when imitating pitch accents of a different (Southern) variety of Italian. In particular, we tested whether Bari Italian (BI) speakers would produce *later* peaks for their native rising L + H^*^ (question pitch accent) in the process of imitating Neapolitan Italian (NI) rising L^*^ + H accents. Also, we tested whether BI speakers are able to modify other phonetic properties (pitch level) as well as phonological characteristics (changes in tonal composition) of the same contour. In a follow-up study, we tested if the reverse was also true, i.e., whether NI speakers would produce *earlier* peaks within the L^*^ + H accent in the process of imitating the L + H^*^ of BI questions, despite the presence of a contrast between two rising accents in this variety. Our results show that phonetic detail of tonal alignment can be successfully modified by both BI and NI speakers when imitating a model speaker of the other variety. The hypothesis of a selective imitation process preventing alignment modifications in NI was hence not supported. Moreover the effect was significantly stronger for low frequency words. Participants were also able to imitate other phonetic cues, in that they modified global utterance pitch level. Concerning phonological convergence, speakers modified the tonal specification of the edge tones in order to resemble that of the other variety by either suppressing or increasing the presence of a final H%. Hence, our data show that intonation imitation leads to fast modification of both phonetic and phonological intonation representations including detail of tonal alignment and pitch scaling.

## INTRODUCTION

A large body of literature has shown that segmental features of speech can either subconsciously or consciously be imitated in the process of online phonetic convergence (Fowler et al., 2003; Pardo, 2006; Nielsen, 2011; *inter alia*). Within the imitation paradigm, various studies within the segmental literature have established that speakers are capable to modulate phonetic detail of their own speech in order to resemble that of speech to which they have previously been exposed (Goldinger, 1998; Nielsen, 2011).

One hypothesis reflecting this phenomenon is that listeners would update their phonetic representations in response to utterances just produced by the interlocutor. The updated phonetic models would hence be responsible for the observed imitation effect. In other words, idiosyncratic elements of how a word was pronounced by the speaker would not be filtered out, which is what is commonly assumed by normalization and abstract representation models (i.e., Gaskell and Marslen-Wilson, 1996). An important factor in the degree of phonetic convergence measured in the spontaneous imitations is word frequency. Goldinger (1998) has first shown that low frequency words are better imitated than high frequency words, and that frequency interacts with amount of exposure to stimulus in predicting degree of imitation (i.e., the amount of phonetic detail being reproduced). This is because low frequency words would be characterized by weaker episodic traces, hence being more prone to a modification in their representation. Note that in an episodic or “exemplar” model, every perceived spoken item leaves a unique memory or “episodic” trace in which detailed phonetic characteristics, such as the voice of the speaker, are stored. However, all these previous studies concern single word imitation, and not sentences nor phrases, and do not address possible modifications relative to the suprasegmental level. In this study we ask whether intonation representations can also be rapidly modified as a result of phonetic and phonological convergence.

Concerning intonation, we know that the intonational features of different languages and language varieties can differ both at the phonological as well as at the phonetic level (Ladd, 1996/2008). Cross-variety (and cross-linguistic) intonational differences at the phonological level concern differences in the inventory of phonologically distinct tunes or pitch accent types, and can either concern their formal properties or the meanings assigned to them (Ladd, 1996/2008; Grice et al., 2005). At a phonetic level, cross-linguistic intonation differences can involve different realizations of an identical pitch accent type, in terms of a number of dimensions specifying both the temporal and melodic characteristics of a tonal target. These dimensions are referred to as *tonal alignment* and *scaling*. Tonal alignment refers to the temporal specification of a fundamental frequency (*f0*) peak or valley relative to the stressed syllable with which the accent is associated (cf. D’Imperio, 2006, 2011 for a review). Specifically, alignment specifies the synchronization of tone targets and specific prosodic units (such as the syllable) or segmental landmarks (such as syllable onset or offset). Within the Autosegmental–Metrical (AM) theory (see Ladd, 1996/2008 for a review), tonal alignment is the main feature determining a pitch accent category.

A few studies have investigated phonological and phonetic aspects of intonation by means of the imitation paradigm. Some researchers have underlined the fact that speakers can successfully and spontaneously reproduce specific intonation features of utterances just heard, such as pitch accent and tonal boundary location (Cole and Shattuck-Hufnagel, 2011) as well as global, phonological aspects of the tune (Braun et al., 2006), such as pitch accent and boundary tone combination (i.e., nuclear contour shape), and this also between dialects of the same language (cf. Romera and Elordieta, 2013 on spontaneous convergence between Catalan and Majorcan Spanish). Braun et al. (2006) have also shown that, when reproducing randomly variable intonation contours, speakers appear to be able to extract linguistically meaningful (i.e., phonological) contrast by concentrating their productions around a limited number of prototypical patterns or “attractors.” In other words, English speakers can only reproduce *f0* contours which are grammatically meaningful, just as in segmental imitation speakers might only be able to imitate phonemes of their own language.

According to another line of work, speakers appear to be able to spontaneously imitate global acoustic prosodic features, such as pitch level and speech rate, of a previously heard utterance, either in absence (Bosshardt et al., 1997) or in presence (Gregory et al., 1997) of direct social interaction. More recently, direct imitation studies have shown that phonological contrastive tonal patterns can be imitated both within (Michelas and Nguyen, 2011 for French) and across varieties of the same language (German, 2012 for English).

Note also that tonal alignment possesses both a phonetic and a phonological dimension. From a phonological point of view, tonal association specifies the head tone within a pitch accent (Grice, 1995), while alignment concerns the phonetic implementation of the temporal specification. In American English, for instance, the different tonal association of L + H^*^ and L^*^ + H results in the fact that peaks are reached earlier within the first than in the second accent type, and this is also true for a number of other languages, such as Neapolitan Italian (NI). In Neapolitan, specifically, the late peak accent (L^*^ + H) characterizes yes/no question contours while the early peak accent (L + H^*^) characterizes narrow focus statements (D’Imperio, 2000). Bari Italian (BI), on the other hand, lacks a specific alignment contrast between two types of accentual rises, though alignment differences can be found both at the individual level, or else concerning other configurations.

Just as other aspects of phonology, such as segmental contrast, both melodic and temporal characteristics of pitch accents have been found to be phonologically contrastive in categorical perception studies. For instance, D’Imperio and House (1997) have shown that fine detail of temporal alignment of pitch peaks can help Neapolitan listeners identify yes-no questions when the *f0* peak of a statement rise-fall is delayed within the stressed syllable. On the other hand, minor alignment differences can be present within the same pitch accent category due to phonetic implementation involving either individual difference (Niebuhr et al., 2011) and/or regional variety (Ladd et al., 2003). House (1997, 2004) offers a review of pitch perception data in different languages showing that differential sensitivity to peak alignment at pitch category boundaries is usually not below 50 ms. Hence, on the basis of perception studies, we will consider any alignment modification below 50 ms to be necessarily phonetic and not phonological in nature, given that a phonological contrast needs to be perceptible (Hume and Johnson, 2001).

Moreover, within a given pitch accent category, tonal alignment differences behave similarly to different Voice Onset Time (VOT) values (cf. Atterer and Ladd, 2004 for proposing this analogy) for the same plosive in the languages of the world. VOT imitation in plosives has been recently explored for American English. In a shadowing task, Nielsen (2011) asked speakers to repeat American English stimuli in which VOT was either artificially shortened or lengthened. Specifically, her data show that sub-phonemic VOT differences can be spontaneously imitated in American English, since participants produced significantly longer VOTs after being exposed to target speech in which voiceless plosives were manipulated so as to have extended VOTs. However, artificially shortened VOT was not successfully imitated in this study, which the author explains through a “selective imitation” process. In other words, imitation would be constrained by a perceptual feedback mechanism ensuring that native phonological contrasts are not endangered through the production of a novel category. This suggests that phonetic detail can be perceived and imitated, but only when it does not compromise pre-existing phonological contrast.

This issue is particularly interesting, in our view, especially in the light of what is known in L2 acquisition and alignment. In fact, among intonation features, tonal alignment appears to be the hardest feature to be acquired/modified by non-native speaker (Arvaniti et al., 1998; Mennen, 2004). In Mennen’s study, Dutch learners of Greek were tested as to their ability to reproduce the exact alignment characteristics of Greek prenuclear rises. While they could not reproduce exact L2 values of peak alignment, they did, however, show an influence in their realization of their (Dutch) L1 alignment contrasts. Hence, an interesting bidirectional influence on tonal alignment realization was revealed by this study.

In this paper, we investigate imitation of both phonetic and phonological aspects of intonation across Italian varieties, specifically in relation to the “selective imitation” process. Here we specifically test if the tonal alignment features of BI tunes can be handled “online” and modified in order to imitate a pragmatically (but not formally) identical tune produced by a speaker of another Southern variety, namely NI. In a follow-up study, we also asked if the opposite is true, i.e., whether NI speakers can imitate BI intonation features.

**FIGURE 1:**
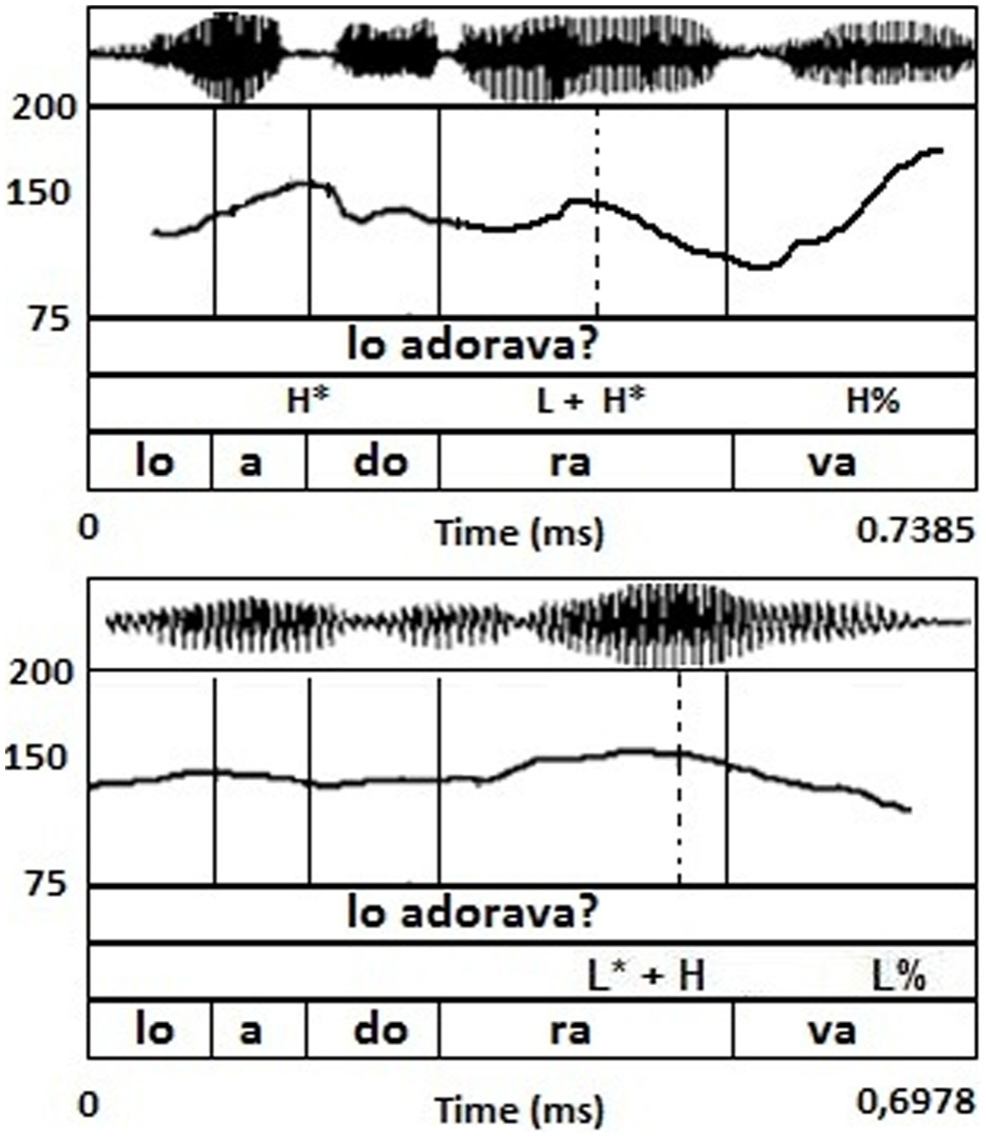
**Yes–no question utterance *Lo adorava?* ‘Did he/she adore it?’ produced by a BI speaker **(upper)** and by the model NI speaker **(lower)**.** The straight line indicates the syllabic boundaries for each word of the sentence; while, the dashed line indicates the alignment point of the nuclear pitch accent (L + H^*^/L^*^ + H).

For the purpose of this study, the selected tunes were all yes/no questions. Our choice was dictated by the fact that the main pitch accent is a LH rise in both Italian varieties under study (Bari and NI, see Grice et al., 2005), though having different association and alignment properties allowing us to test phonetic convergence against the selective imitation hypothesis, as detailed below. Note that in Italian yes/no questions lack a specific morpho-syntactic marking and can be solely expressed by intonation. In particular, the primary cue to interrogation in the Southern varieties is a rising LH pitch accent (L + H^*^ in BI and L^*^ + H in Neapolitan, cf. D’Imperio, 2000; Grice et al., 2005), immediately followed by a phrasal fall or a rise. The main cross-variety difference is that the accentual peak is reached around the middle of the accented syllable in BI, while it is reached later (at the offset of the nuclear syllable) in Neapolitan (D’Imperio, 2002). This alignment contrast is shown in **Figure 1**.

Note that BI and NI yes-no question contours differ also as to the phonological specification of the yes–no intonation contour. Specifically, while prototypical BI question contours show a IP-final H% rise, prototypical NI questions show a IP-final L% fall (D’Imperio, 2000, 2002). Moreover, a paradigmatic difference between BI and NI concerns the inventory of contrasting pitch accents. In fact, BI does not show a contrast between two rising pitch accents, since it only possesses L + H^*^ (Grice et al., 2005). This predicts that the exact alignment of the H in the BI L + H^*^ accent might be more variable and easily modifiable given that no perceptual contrast would be endangered as a result of such a modification. On the other hand, NI possesses both L + H^*^ and L^*^ + H, which predicts that Neapolitan would show a resistance to anticipate the H target in questions in order to avoid contrast erasure. If, in line with the selective hypothesis, imitation is achieved by selecting only variants for which contrast is not endangered, we expect Bari speakers to be more successful in imitating the Neapolitan tunes, while Neapolitans would behave differently. In other words, given that BI speakers do not possess a contrast between two accentual rises, we postulate that the alignment difference can only be perceived and reproduced in terms of a variant of the native L + H^*^ rise. We hence predict that Bari speakers would produce later peaks for the BI L + H^*^ question pitch accent, in the process of imitating Neapolitan L^*^ + H. We also tested whether NI speakers would be capable of anticipating the H target within the L^*^ + H in order to resemble the BI L + H^*^, which would not support the selective imitation hypothesis. This is because an earlier rise could resemble the Neapolitan L + H^*^, which is employed for statements.

Another factor that might influence intonation convergence is lexical frequency. For instance, duration has been found to be shorter for higher than lower frequency words (Goldinger, 1998; Bybee, 2001; Aylett and Turk, 2006). Given that tonal alignment is a temporal phenomenon, in that it is timed relative to segmental landmarks, it might be sensitive to duration variation within the syllable. Hence, an issue investigated here is whether the observed alignment effects might be due to frequency-induced duration differences or whether they might be directly influenced by lexical frequency itself. This is why alignment was measured both in absolute terms and proportionally, relative to the duration of the stressed syllable (see Measurements below).

We also tested whether both BI and NI speakers would reproduce global phonetic features such as pitch level (i.e., the overall pitch level of the utterance as produced by the model NI/BI speaker). Finally, structural properties of the tune, such as the presence of a specific boundary tone at the right edge of the Intonation Phrase (IP), were also specifically analyzed in order to verify whether both phonetic and phonological cues to intonation can be imitated. Specifically, we tested whether BI speakers would produce a smaller number of H% edge tones while imitating Neapolitan, since prototypical yes/no questions in NI lack a final H% rise. The opposite was tested for NI imitation productions. Our results will be discussed in the light of recent convergence studies in the segmental domain.

## MATERIALS AND METHODS

### PARTICIPANTS

Fourteen native BI speakers (eight females and six males) participated in the main experiment and were recruited from the graduate population of Bari University. In a follow-up study, six speakers of NI were also recruited (three females and three males) from the graduate population of Aix-Marseille University. The NI participants were carefully chosen from a group of Italians recently arrived in France and with minimal knowledge of French. All participants reported normal hearing and their age varied between 25 and 35. They were all paid for their participation. Participants agreed with the experimental procedure and signed an informed consent. Both experiments were conducted under ethical conditions.

### PROCEDURE

In the main experiment, recordings were performed in a sound-attenuated room at the University of Bari, while for the follow-up study NI speakers were recorded in the sound-proof booth of the *Laboratoire Parole et Langage* in Aix-en-Provence. Subjects were recorded in two separate tasks, specifically a Baseline and an Imitation task, which were run in sequence within the same recording session. The Baseline always preceded the Imitation session, so that convergence could be assessed relative to the default, base pronunciation of each speaker. Each session typically lasted 15 min. During the Baseline task, target sentences were presented one at a time on a computer screen. Each target sentence was preceded by another sentence, which served the purpose of setting up the context for the utterance to be produced. Subjects were instructed to read aloud only the second sentence by speaking into a microphone (HD 60) using their native intonation (either BI or NI). The purpose of the Baseline task was to obtain a reference production for each variety. Each target sentence was produced three times, in a random fashion, with a yes/no question intonation.

During the Imitation task, participants were told that they would be listening to a recording of a speaker using an “unfamiliar dialect” and that they should try to imitate his pronunciation as closely as possible while repeating the sentence just heard (see also German, 2012). The same instructions were given for both experiments presented here. Explicit imitation instructions were preferred to a simple shadowing task with the aim of maximizing the effects, given that it appears that alignment features cannot be easily modified in L2 acquisition (Mennen, 2004). Subjects were not given any information about the variety that they were to imitate. Stimuli were presented using a PowerPoint presentation. Each subject was seated in front of a computer with a head-mounted microphone and headphones.

In the follow-up study, corpus and procedure were the same as in the main experiment, though this time participants were Neapolitan speakers and the model was a Bari speaker.

### MATERIALS

The total set of experimental materials was composed as follows. For the main experiment, 20 target words (10 low frequency and 10 high frequency words ^*^ 3 repetitions ^*^ 2 sessions); for the follow-up experiment 18 target words (8 low frequency and 10 high frequency words ^*^ 3 repetitions ^*^ 2 sessions). This difference in the corpus is due to the fact that for the follow-up we chose as a model speaker a BI participant who produced two sentences which did not show the prototypical BI question pattern^1^. The corpus was the same in the Baseline and the Imitation task, though in the Imitation Task the model speaker was either a NI (for the main experiment) or a BI speaker (for the follow-up). The NI model was a male native speaker of NI, whose age was 38, while the model BI speaker was a 34-year old female.  show, respectively, mean values for both NI and BI model speaker productions.

**Table 1:** Average H alignment (expressed as a percentage relative to the accented syllable duration), global pitch level (in semitones), and tonal specification of the IP-final edge tone for all the utterance produced by the model Bari Italian (BI) speaker.

Word frequency	H alignment (%)	Pitch level (st)	IP-final L% (%)	IP-final H% (%)
High	57	91.8	0	100
Low	53.2	91.5	0	100

**Table 2:** Average H alignment (expressed as a percentage relative to the accented syllable duration), global pitch level (in semitones) and tonal specification of the IP-final edge tone for all the utterance produced by the model Neapolitan Italian (NI) speaker.

Word frequency	H alignment (%)	Pitch level (st)	IP-final L% (%)	IP-final H% (%)
High	71.7	81.41	100	0
Low	76.7	81.30	100	0

Lexical frequency was determined on the basis of the CLIPS database (De Mauro et al., 1993), with the thresholds for low- and high-frequency words being, respectively, 300 occurrences per million for high-frequency words and five occurrences per million for low-frequency words. Target stressed syllables were always open (i.e., *manGIAva*, *anDAva* “(he/she) ate, (he/she) went”), to control for alignment effects, and were penultimate within the word. All target words were simple past verbs (all three-syllabic and with penultimate stress) preceded by a pronominal particle (i.e., *Ci veniva?,* litt. “Would he come by?,” *Lo adorava?*, “Did he adore it?”). The expected pitch accent pattern was a nuclear rising LH accent (either L^*^ + H for Neapolitan or L + H^*^ for BI) on the target word (see Appendix A for more information about the corpus). All utterances were produced with yes/no question intonation by both model speakers. The productions of 14 BI speakers resulted in a total of 1680 sound files, while the production of the six NI speakers yielded 648 observations. From these, for both experiments, a total of 222 (9.5%) productions had to be eliminated for technical errors, so that a total of 2099 (1485 files for the main experiment and 614 files for the follow-up experiment) were included in our analyses.

Note that, as expected, the proportional alignment reported in **Tables 1** and **2** shows later alignment for the Neapolitan (**Table 1**) than the BI model speaker (**Table 2**), with a difference of around 20%.

### MEASUREMENTS

Utterances were automatically extracted and saved as separate files through PRAAT (Boersma and Weenink, 1992/2010). Subsequently, each sound file was segmented with the software SPPAS (Bigi, 2012) into words, syllables, and phonemes. Intonational features of the recorded utterances were tagged by hand by the second author. Specifically, we labeled the target H tone within the nuclear stressed syllable, and the presence of a H% vs. a L% boundary tone at the end of the IP. The accentual H target was often realized as an *f0* peak within the region of the accented syllable (i.e., the penultimate syllable in the utterance) and was hence automatically labeled. When a plateau (i.e., a series of points having fairly level *f0* values) was identified, the last *f0* point was selected as the H peak location (see D’Imperio, 2000). The overall pitch level for the utterance was also measured in semitones^2^.

The statistical significance of the results was tested using a series of mixed models by means of the R-environment (R Development Core Team, 2012). The models were separately run on three dependent variables: the alignment of the H peak, the pitch level of the entire utterance and the presence of a H% vs. L% boundary tone. The alignment of the H peak was calculated first, by (1) using an absolute value of the distance between the H target and a specific landmark in the segmental string (i.e., the syllable onset); subsequently, by (2) a proportional measure relative to the duration of the accented syllable. In fact, though absolute alignment is the most widely employed method in intonational studies, proportional alignment allows to better control for possible frequency effects on segmental duration (Goldinger, 1998), which in turn could affect alignment results (cf. Prieto et al., 1995; Petrone and Ladd, 2007). Tonal alignment and pitch level served as continuous dependent variables in standard linear mixed models. Logit models with mixed effects were applied to account for the presence of H% vs. L% at the end of the utterance. Regional variety of the imitators (Bari/Neapolitan), Task (Baseline/Imitation), and Word Frequency (low/high) were included as fixed factors. For pitch level, the factor Gender (male/female) was added since females have higher fundamental frequency than males. The models had a full random effects structure, i.e., with all possible random intercepts and random slopes. Since the full models showed some over-parameterization, backward elimination based on graphical inspections and on likelihood-ratio tests was used to decide which random terms and interactions among fixed factors should be retained in the models. Likelihood-ratio tests (Baayen, 2008) were run comparing full models (e.g., which contained an interaction) with simpler ones (e.g., without that interaction).

Note that standard mixed models do not provide *p*-values when random slopes and intercepts are correlated. Given the large dataset, we hence assumed that a fixed factor was significant if its t value would be greater than 2 (Baayen et al., 2008). On the other hand, for our logit models the cutoff point for significance was set at *p* < 0.01.

## MAIN EXPERIMENT RESULTS

### TONAL ALIGNMENT

**FIGURE 2:**
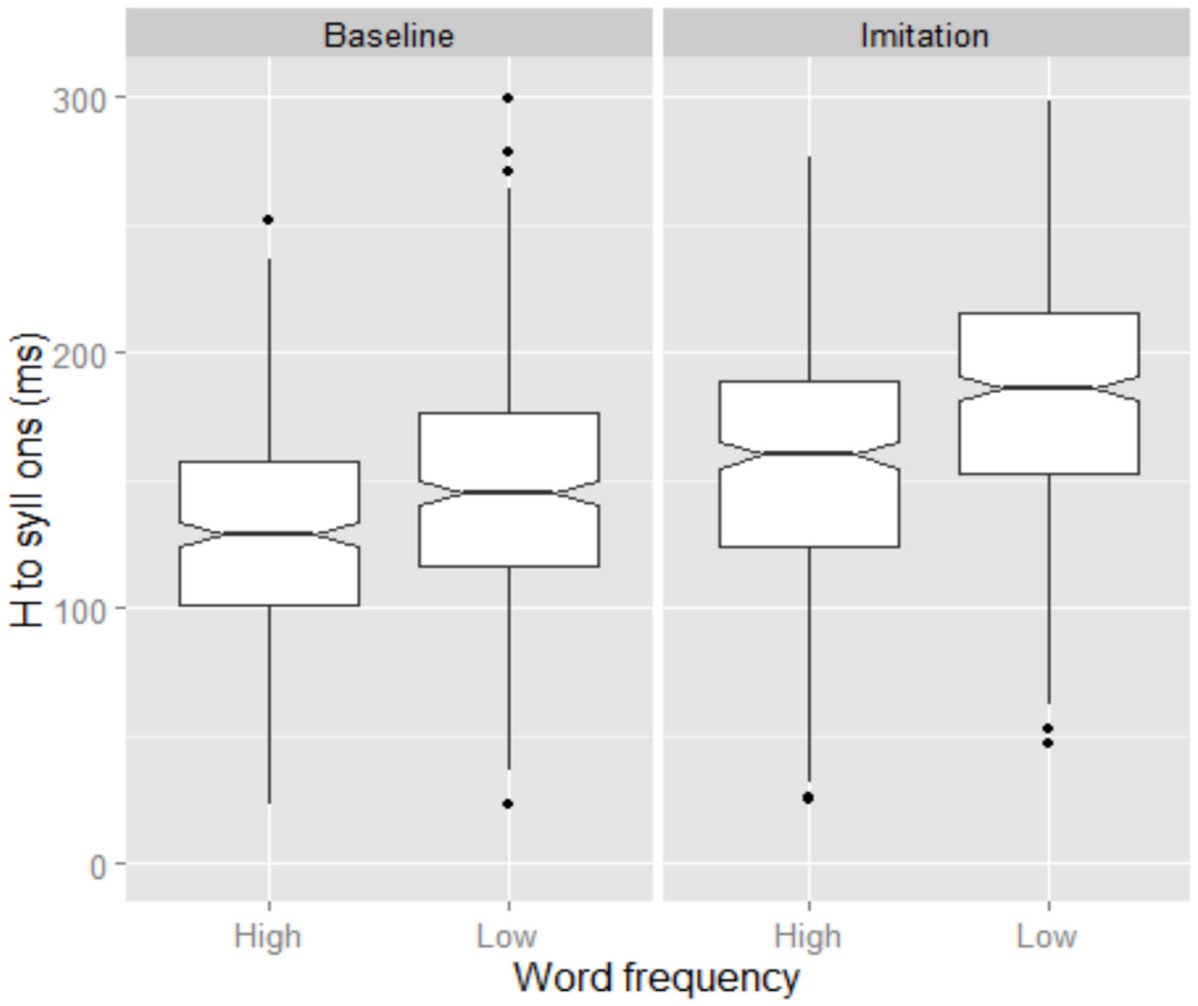
**Boxplots for H alignment relative to syllable onset produced by BI speakers.** Data are shown across Word frequency and Task. The 0 level in the y-axis indicates syllable onset. The notch indicates the 95% confidence interval for comparing medians.

In **Figure 2**, tonal alignment across Word frequency is displayed for both the Baseline and the Imitation tasks. Here, tonal alignment was calculated relative to the syllable onset. The figure shows that the H target was generally aligned earlier in the Baseline than in the Imitation task, as predicted by our main hypothesis. The statistical analysis confirmed a significant effect of Task (β = -0.02, SE = 0.008, *t* = -2.36). Furthermore, **Figure 2** shows that H target alignment was later in low than in high frequency words, in both the Baseline and the Imitation task (β = 0.029, SE = 0.008, *t* = 3.39). Note though that the frequency effect might be an epiphenomenon of differences in accented syllable duration. In fact, those syllables were significantly longer in low than in high frequency words, in both the Baseline (high = 240 ms; low = 257 ms) and the Imitation (high = 230 ms; low = 257 ms) tasks (β = 0.02, SE = 0.002, *t* = 8.94).

**FIGURE 3:**
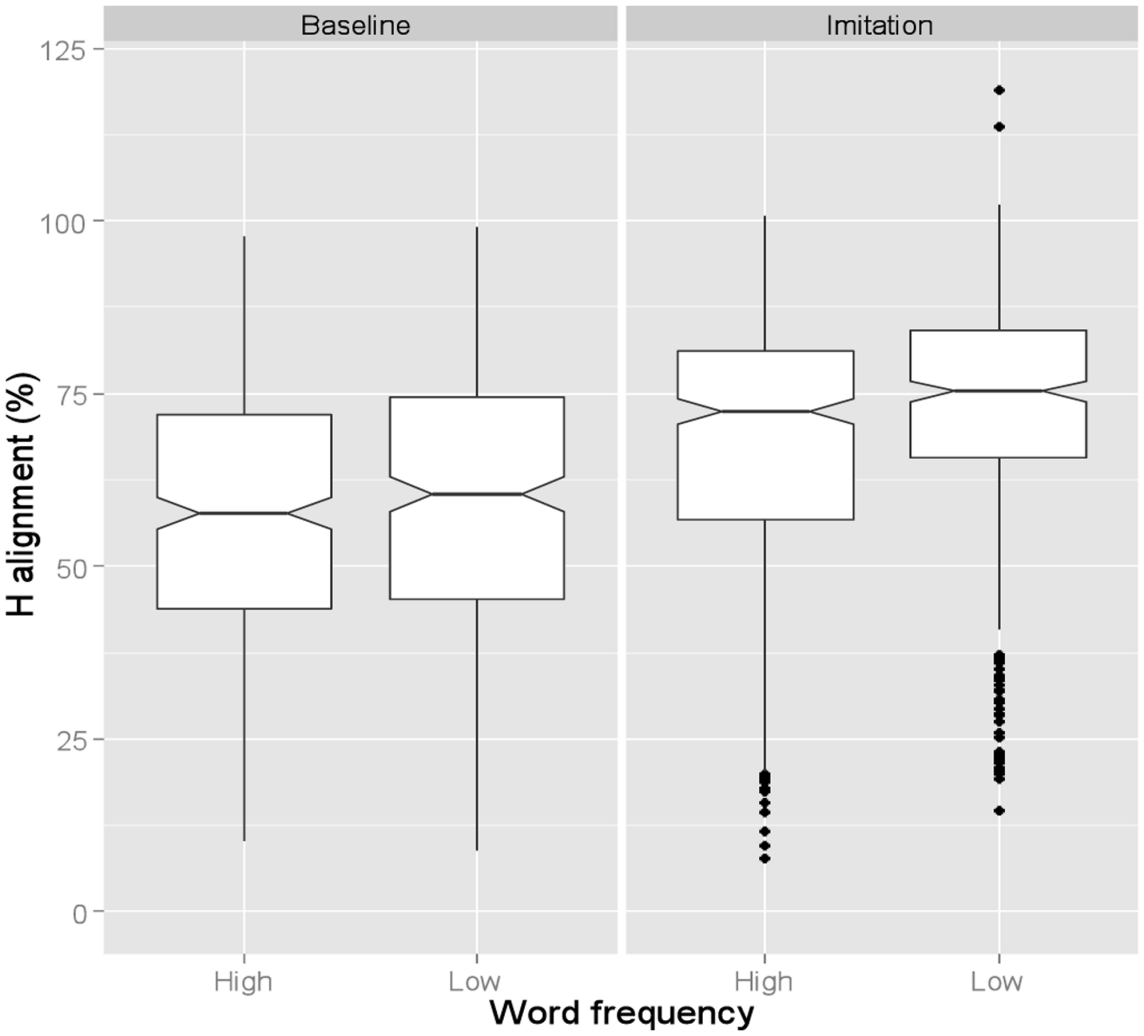
**Boxplots for proportional H alignment produced by BI speakers.** Data are shown across Word frequency and Task. The 0 level in the y-axis indicates syllable onset. The notch indicates the 95% confidence interval for comparing medians.

Hence, to verify whether alignment differences reflect a genuine effect of Word Frequency, we also calculated tonal alignment as a proportion of the accented syllable duration (**Figure 3**). Similar to **Figure 2**, **Figure 3** shows that the H target was again aligned earlier in the Baseline (57.4%) than in the Imitation (70.3%) task. The effect of Task was also significant (β = -10.06, SE = 3.5, *t* = -2.86). **Figure 3** also shows, on the other hand, that, when alignment is calculated proportionally, the Word Frequency effect is no longer found in the Baseline task (low = 58.1%; high = 56.1%) though it is still visible in the Imitation task, where the alignment was later in low than high frequency words (low = 72.6%; high = 67.9%). Word Frequency was indeed significant only for the Imitation task (β = 4.71, SE = 2.2, *t* = 2.14).

### PITCH LEVEL

**FIGURE 4:**
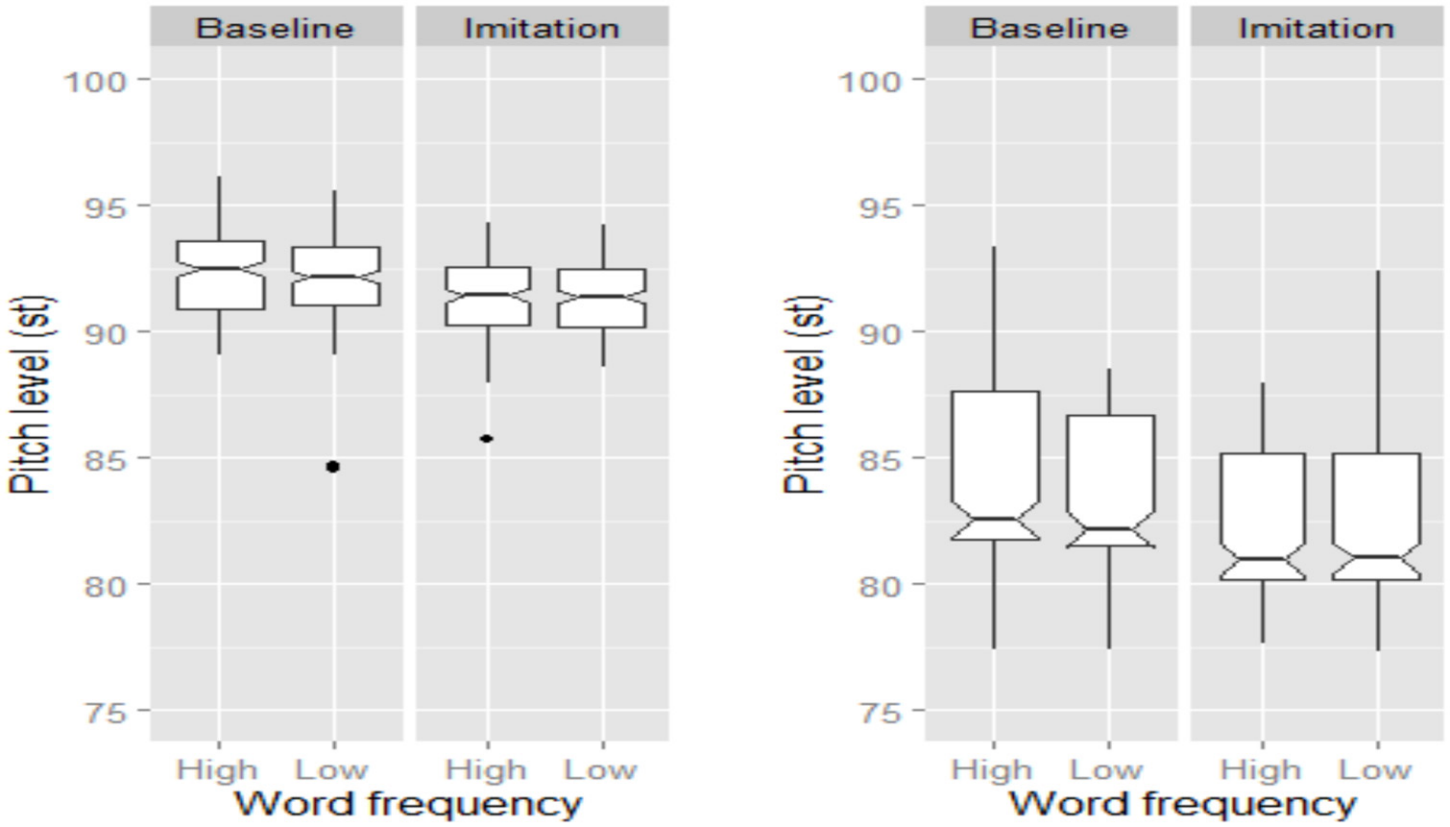
**Boxplots for pitch level (in st) across Word Frequency and Task for BI speakers.** Data for female and male speakers are separately plotted in the **left** and **right** panel, respectively. The notch indicates the 95% confidence interval for comparing medians.

As shown in **Figure 4**, both female and male speakers successfully lowered their global pitch level when imitating the model talker. The statistical model showed a significant effect of Task, with pitch level being higher in the Baseline than in the Imitation task (β = 0.97, SE = 0.08, *t* = 12.07). Note that male speakers’ utterances presented a lower pitch level than those produced by female speakers (β = -6.81, SE = 1.68, *t* = -4.04). However, there was no main effect of Word Frequency nor significant interactions.

### TONAL SPECIFICATION

Apart from phonetic features, speakers significantly changed structural aspects of the tune while imitating the model speaker, both for BI (**Table 3**) and NI speakers (**Table 4**). In the Baseline, BI speakers mostly produced a rising movement to a high edge tone (H%) and, in a smaller percentage, a rising movement to a mid tone target !H% (see also Gili Fivela et al., to appear), which is an allophonic variant of H% in this variety. In the Imitation task, they significantly produced a majority of L% edge tones, typical of the NI yes/no question tune, and, consequently, a smaller number of H% at the end of the question utterance (β = 1.34, SE = 0.17, *z* = 7.57, *p* < 0.01). Note that no actual L% was produced in the Baseline by BI Italians. An example of !H% production is reported in Appendix C. Note that NI speakers (**Table 4**), different from the model NI speaker, already produced a high number of H% in the Baseline (see also Discussion for an account of this difference), probably due to a reading style effect.

**Table 3:** Percentage and number of occurrence (in parentheses) of !H%, H% and L% boundary tones in the Baseline and Imitation task for BI speakers.

Baseline	Imitation
!H% = 32.5% (248)	L% = 61% (516)
H% = 67.5% (439)	H% = 39% (282)


**Table 4:** Percentage and number of occurrence (in parentheses) of H% and L% boundary tones in the Baseline and Imitation task for NI speakers.

Baseline	Imitation
L% = 18% (55)	L% = 7% (21)
H% = 82% (248)	H% = 93% (290)


### FOLLOW UP – EXPERIMENT RESULTS

**FIGURE 5:**
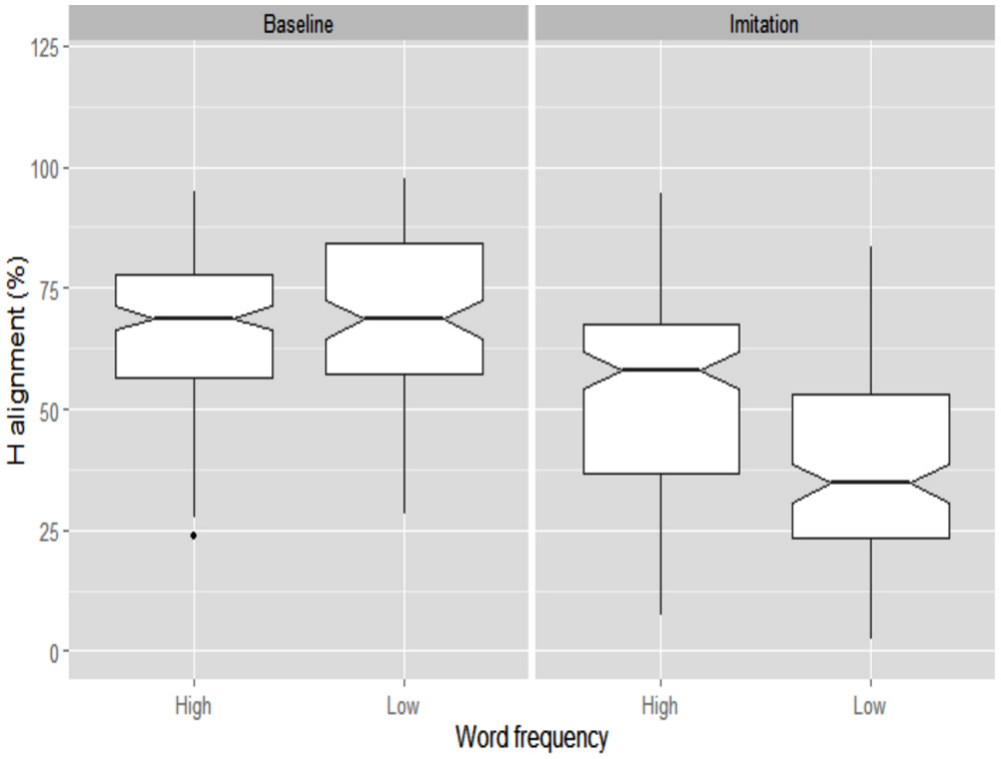
**Boxplots for H alignment across Word Frequency and Task for NI speakers.** The 0 level in the y-axis indicate syllable onset. The notch indicates the 95% confidence interval for comparing medians.

In order to test the selective imitation hypothesis, we carried out a follow-up study with NI speakers imitating a BI model speaker, given that NI shows a contrast between two accentual rises having different association and alignment properties. Generally speaking, Neapolitan speakers were able to imitate tonal alignment, pitch level, and edge tone specification of BI utterances. Note that only proportional alignment was analyzed so as to avoid the duration confound related to Word Frequency (see Tonal Alignment above). **Figure 5** shows that proportional tonal alignment in the Baseline task was later (average across word frequency: 67.4%)^3^ than in the Imitation task (45.5%), indicating that NI speakers successfully imitated the early alignment of BI L + H^*^ nuclear accents. The Task effect was significant (β = 14.03, SE = 5.24, *t* = 2.67). The significant interaction between Task and Variety indicates that the difference between Baseline and Imitation was larger for NI than for BI imitators (β = 24.03, SE = 6.18, *t* = 3.89). As in the main experiment, Word Frequency was significant only in the Imitation task (β = -16.52, SE = 2.56, *t* = -6.44). However, the direction of the effect was, as expected, reversed relative to the one found in the main experiment, with the pitch peak being aligned earlier in low (36.37%) than in high (56.01%) frequency words. This is coherent with the fact that the direction of the alignment difference being imitated by the participants was the opposite in the two studies, but it also further supports that the frequency effect is not due to specific phonetic properties of the words employed; otherwise the direction of the effect would have been the same in the two studies.

**FIGURE 6:**
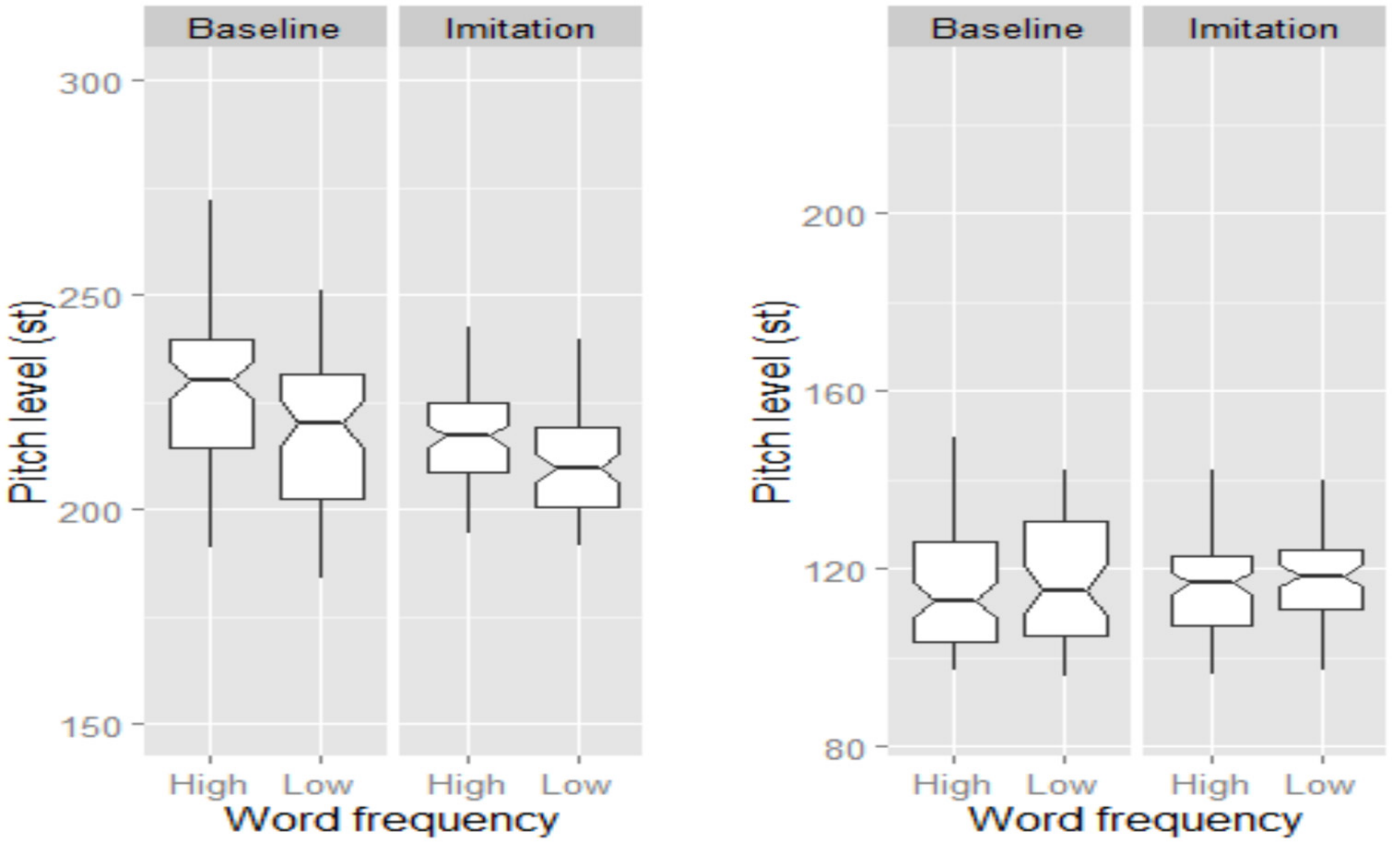
**Boxplots for pitch level (in st) across Word frequency and Task.** Data for female and male speakers are separately plotted in the **left** and **right** panel, respectively. The notch indicates the 95% confidence interval for comparing medians.

An effect of Task and Word Frequency was also found for pitch level, but only for female speakers (**Figure 6**), whose pitch level was higher in the Baseline than in the Imitation task, indicating that they significantly lowered their pitch level to imitate the model talker (β = -0.55, SE = 0.17, *t* = -3.21). Moreover, low frequency words were characterized by lower pitch level than high frequency words (β = -0.55, SE = 0.17, *t* = -3.21). Finally, there was no significant difference in *tonal specification* across the experimental variables, though NI speakers appeared to slightly increase the number of H% occurring at the end of the imitated utterances. Lack of significance might by due to the high number of H% already being produced in the Baseline, which we explain through a reading style effect (see also Discussion section).

### GENERAL DISCUSSION

Our results show that phonetic detail of intonational structure such as tonal alignment can be successfully imitated across two Southern varieties of Italian, i.e., BI and NI. Specifically, BI speakers were able to displace their H peaks later in order to imitate the model NI speaker in the Imitation phase, while NI speakers were able to shift H peaks earlier to imitate the model BI speaker. This is surprising given that details of tonal alignment are not easily learned and reproduced by L2 speakers (Mennen, 2004) and might be taken to suggest higher sensitivity to phonetic detail within dialects of the same language than across languages. Given that the effect was significant in both varieties, the selective imitation hypothesis does not seem to hold true for intonational contrast. In other words, despite the presence of two contrastive rising accents (L + H^*^ and L^*^ + H), NI speakers appear to potentially compromise yes/no question tune identity by moving the H peak earlier. This is not surprising given that alignment might have been compensated by the production of a larger number of final H% (93.2%) in the Imitation phase, that might have helped marking question-hood and hence limiting ambiguity.

Note also that later alignment within the native L + H^*^ might lead to new category formation, that is, leading to the production of a L^*^ + H, existing in NI but not in BI. In line with selective imitation, we predicted that NI speakers would avoid alignment modification in producing rising pitch accents, since NI shows a contrast between L + H^*^ and L^*^ + H. This was not the case, since NI speakers did not suppress H alignment anticipation to prevent contrast erasure. This is taken to suggest that phonetic convergence for intonation detail might not be as selective as for segmental contrast, such as VOT differences (Nielsen, 2011). On the other hand, intonation meaning is not based on nuclear pitch accent category alone, but also on edge tone identity, according to a compositional account. Hence, question vs. statements identification might be preserved by the use of features other than pitch accent category, such as final contour. In other words, Neapolitan speakers might give more functional weight to a final, non-native H%, as a question marker, than to the nuclear pitch accent whose alignment features are prone to modify.

An unexpected result was that the magnitude of the realignment was higher for NI than for BI speakers, since in the follow-up experiment speakers advanced their peaks in a more substantial way relative to the temporal shift in the BI experiment. We speculate that this might be due to intonation transfer in NI, in which an early H^*^ + L accent, with a very early peak, is available in the tonal inventory. In other words, while for NI the alignment imitation might have led to phonological assimilation and intonation transfer, for BI it might be the result of a mere phonetic convergence effect.

Also, when imitating the model speaker, BI speakers were able to reproduce global prosodic/phonetic cues, in that they lowered utterance pitch level in order to imitate the level of the model speaker, independent of gender. In the NI study, on the other hand, the pitch level shift was significant only for female speakers. The difference between the BI and the NI results might, however, be due to the higher pitch level of the model speaker, which might have prevented male speakers to perceive and hence readjust their own global level.

Within the segmental literature, there is ample evidence for spontaneous phonetic convergence at the segmental level (see Pardo, 2006 *inter alia*). However, no study reports at the same time phonetic and phonological convergence for intonation. Our general phonetic results, both for tonal alignment and pitch level adjustments, appear to go counter to recent findings for American English intonation convergence. A study by Cole and Shattuck-Hufnagel (2011), for instance, supports the view that only structural, phonological aspects of the intonation of a model speaker might be successfully imitated. Specifically, in their study American English speakers correctly reproduced pitch accent (especially for nuclear pitch accents) and tone boundary placement from a previously heard spontaneous utterance, while they were less accurate in terms of imitated phonetic detail.

A possible explanation for the difference between our results and those reported by Cole and Shattuck-Hufnagel (2011) might reside in the different instructions given to participants. In fact, in our study (as in German, 2012), participants were asked to explicitly imitate the voice of the model speaker. Focusing speakers’ attention on the model speaker’s voice might have resulted in decreased imitation of structural aspects, such as boundary tone type as well as pitch accent placement, and increased attention on phonetic aspects of the utterances heard. Note though that in our study speakers were able to rapidly reproduce both structural and phonetic aspects of previously heard utterances. Another potential cause for the difference in our results and the previous ones is that the utterances of our study were short and controlled in the laboratory, while in Cole and Shattuck-Hufnagel’s (2011) study stimuli were excised from a spontaneous speech corpus.

A notable effect was also that both tonal alignment and pitch level imitation were significantly stronger for low frequency words (though this was true only for female speakers for the pitch level results in the follow-up study). This effect was independent of absolute word duration, and was present only in the Imitation phase for both BI and NI speakers, when alignment was calculated in proportional terms. We account for this unexpected result in terms of a heightened difficulty in rapidly adapting the new alignment values to syllable or word duration in the Imitation phase in rare items. If the frequency effect were merely due to specific word phonetic properties of the words employed, the direction of the effect should have been the same in the two experiments. Instead, while low frequency words were associated to later alignment values in the BI study, the opposite was true for the NI study. In other words, the effect is driven by the categorical indexing value of the alignment feature, and not by a surface phonetic effect. This also lends further support to the idea that fine phonetic detail is more perceivable for items possessing weaker episodic traces (Goldinger, 1998). This finding has implications for exemplar models of intonation (Calhoun and Schweitzer, 2012), in that temporal and melodic aspects of intonation, just as other phonetic detail pertaining to segmental structure, might be more resistant to rapid modification in items possessing stronger lexical specifications.

Note also that previous direct imitation studies show that speakers are able to extract linguistically meaningful intonational contrasts by concentrating their productions around a limited number of “attractors” (Braun et al., 2006). In our study, as for phonological aspects of the tune, participants were also quite successful imitators. In fact, BI speakers produced a greater number of L% edge tone in place of H% (typical of BI questions) at the end of the imitated utterances. On the other hand, NI speakers only showed a trend in producing a greater number of H%, as opposed to L%, as question edge tone markers, since the result was not significant. This was due to the presence of an already important number of H% in the baseline for NI speakers, due to a reading style effect that has already been reported in the literature for other Southern varieties (Grice et al., 1997). A final rising edge tone is typical of question contours in Tuscan and Northern varieties of Italian, which are usually associated with higher socio-economic status. Hence, when asked to be recorded, Southern speakers have a tendency to apply the prestigious final H% edge tone. They nonetheless manage to maintain the nuclear pitch accent category and alignment characteristics of their native dialect.

In conclusion, in line with the results shown in Michelas and Nguyen (2011), intonation imitation appears hence to be fast and quite robust. Moreover, our study shows that intonation imitation can also lead to phonetic convergence in tonal alignment detail, at least across varieties of the same language. In fact, speakers appear to be able to perceive, retain and readily use phonetic detail beyond phonological contrast (since rise alignment is not contrastive in BI), suggesting that contrastive phonological elements might not constitute a complete description of mental representation of intonation (see also Braun et al., 2006). Finally, similar to findings for acoustic/segmental features, internal phonetic representations of intonation appear to be readily updated even in absence of direct social interaction. In other words, fine tuning of phonetic cues (such as tonal alignment) did not prevent speakers to imitate more global aspects of the tune itself, such as global pitch level as well as the presence of either a rising or a falling boundary tone. To sum up, our results show for the first time the existence of both phonological and phonetic imitation in intonation, pointing to the unexpected capability of modifying peak alignment in a way to resemble the model speaker’s intonation even when this might result in potential confusion with an existing pitch accent category.

## Conflict of Interest Statement

The authors declare that the research was conducted in the absence of any commercial or financial relationships that could be construed as a potential conflict of interest.

## Footnotes

1 Note that this was yet the best speaker in the corpus previously recorded given the prototypicality of his tonal patterns in most of the utterances produced (we chose the model speaker from the set of prerecorded speakers of Experiment 1, from the Baseline productions).

2 Pitch level was calculated as the median log transformed *f0* value for the utterance (De Looze and Hirst, 2008). This is because the median, and not the mean, is a more robust measure for pitch level.

3 Note that the average alignment value obtained in the Baseline is lower than the one reported for the model NI speaker employed in Experiment 1. This may be due to the presence of inter-individual variability that has already been reported in the literature (see Introduction above).
