# Supplementary material for: Phonetic and phonological imitation of intonation in two varieties of Italian
Source: Front Psychol. 2014 Nov 4;5:1226. doi: 10.3389/fpsyg.2014.01226 (PMC4219553; doi:10.3389/fpsyg.2014.01226)
Supplement: Supplementary file 1 [file Data_Sheet_1.DOCX]

**APPENDIX A**

**STIMULI FOR MAIN EXPERIMENT**

For each sentence the target words were simple past verbs (in Italian all of these verbs are three-syllabic with penultimate stress) preceded by a pronominal particle. Stressed syllables, in capital letter, were always open and penultimate within the word.

| **Frequency** | **Items** | **Context** | **Phrase** |
| --- | --- | --- | --- |
|  |  |  |  |
| H | 1 | Cosa faceva? | Lo manGIAva? |
| H | 2 | Cosa faceva? | La prenDEva? |
| H | 3 | Cosa faceva? | Lo veDEva? |
| H | 4 | Cosa faceva? | Lo venDEva? |
| H | 5 | Cosa faceva? | Gli parLAva? |
| H | 6 | Cosa faceva? | Ci veNIva? |
| H | 7 | Cosa faceva? | Lo chiaMAva |
| H | 8 | Cosa faceva? | Lo troVAva? |
| H | 9 | Cosa faceva? | La scriVEva |
| H | 10 | Cosa faceva? | Ci anDAva |
| **Frequency** | **Items** | **Context** | **Phrase** |
|  |  |  |  |
| L | 1 | Cosa faceva? | Lo molLAva? |
| L | 2 | Cosa faceva? | Lo scaVAva? |
| L | 3 | Cosa faceva? | Lo abbiNAva? |
| L | 4 | Cosa faceva? | Lo esiBIva? |
| L | 5 | Cosa faceva? | La indoviNAva? |
| L | 6 | Cosa faceva? | Lo adoRAva? |
| L | 7 | Cosa faceva? | Lo alleGAva? |
| L | 8 | Cosa faceva? | La baGNAva? |
| L | 9 | Cosa faceva? | Lo graDIva? |
| L | 10 | Cosa faceva? | Lo droGAva? |

**APPENDIX B**

**STIMULI FOR FOLLOW-UP EXPERIMENT**

For each sentence the target words were simple past verbs (in Italian all of these verbs are three-syllabic with penultimate stress) preceded by a pronominal particle. Stressed syllables, in capital letter, were always open and penultimate within the word.

| **Frequency** | **Items** | **Context** | **Phrase** |
| --- | --- | --- | --- |
|  |  |  |  |
| H | 1 | Cosa faceva? | Lo manGIAva? |
| H | 2 | Cosa faceva? | La prenDEva? |
| H | 3 | Cosa faceva? | Lo veDEva? |
| H | 4 | Cosa faceva? | Lo venDEva? |
| H | 5 | Cosa faceva? | Gli parLAva? |
| H | 6 | Cosa faceva? | Ci veNIva? |
| H | 7 | Cosa faceva? | Lo chiaMAva |
| H | 8 | Cosa faceva? | Lo troVAva? |
| H | 9 | Cosa faceva? | La scriVEva |
| H | 10 | Cosa faceva? | Ci anDAva |
| **Frequency** | **Items** | **Context** | **Phrase** |
|  |  |  |  |
| L | 1 | Cosa faceva? | Lo molLAva? |
| L | 2 | Cosa faceva? | Lo abbiNAva? |
| L | 3 | Cosa faceva? | Lo esiBIva? |
| L | 4 | Cosa faceva? | Lo adoRAva? |
| L | 5 | Cosa faceva? | Lo alleGAva? |
| L | 6 | Cosa faceva? | La baGNAva? |
| L | 7 | Cosa faceva? | Lo graDIva? |
| L | 8 | Cosa faceva? | Lo droGAva? |

**APPENDIX C**

**
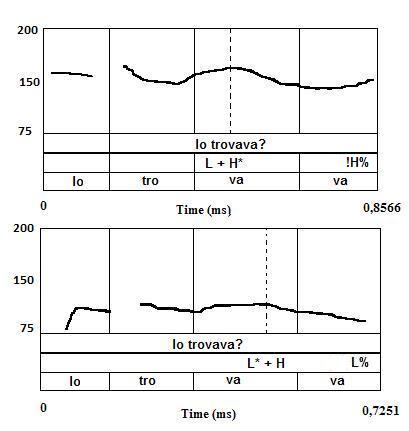
**

Yes-no question utterance *Lo trovava?* ‘Did he/she find it?’ produced by a BI speaker (upper), with a final !H%, and by the model NI speaker (lower), with a final L%. The straight line indicates the syllabic boundaries for each word of the sentence; while, the dashed line indicates the alignment point of the nuclear pitch accent (L+H*/L*+H).
